# Effects of COVID-19 Lockdown on Physical Performance, Sleep Quality, and Health-Related Quality of Life in Professional Youth Soccer Players

**DOI:** 10.3389/fspor.2022.875767

**Published:** 2022-06-13

**Authors:** Jil Keemss, Johanna Sieland, Florian Pfab, Winfried Banzer

**Affiliations:** ^1^Department for Prevention and Sports Medicine, Institute of Occupational, Social and Environmental Medicine, Goethe University, Frankfurt, Germany; ^2^Department of Sports Medicine, Institute of Sport Sciences, Goethe University, Frankfurt, Germany; ^3^Technical University of Munich, Munich, Germany; ^4^Medzentrum Residenz, Munich, Germany; ^5^Eintracht Frankfurt Fußball AG, Frankfurt, Germany

**Keywords:** professional soccer players, adolescents, COVID-19, home training, lockdown, physical performance, sleep quality, health-related quality of life

## Abstract

**Background:**

In March 2020, the COVID-19 outbreak led to the declaration of a pandemic. The accompanying restrictions on public life caused a change in the training routines of athletes worldwide. The present study aimed to investigate the effects of a 13-week supervised home training program on physical performance, sleep quality, and health-related quality of life in professional youth soccer players during the first COVID-19 lockdown in Germany.

**Methods:**

Eight professional soccer players (age range 16–19; height: 1.81 ± 0.07 m; body weight: 72.05 ± 6.96 kg) from a Bundesliga team in Germany participated in this study. During the lockdown, they trained 5–6 days per week with home-based training plans and were monitored via tracking apps and video training. To determine the effects of home training, measurements were taken before (March 2020) and after (June 2020) the home training period. Bioelectrical impedance analysis was used to determine body composition, and an isokinetic strength test and a treadmill step test, including lactate measurements, were used to measure physical performance. Two questionnaires were responded to in order to assess health-related quality of life [Short-Form 36 Health Survey (SF-36)] and sleep quality (Pittsburgh Sleep Quality Index).

**Results:**

When comparing measurements before and after the home training period, we observed significant increases in the following variables: body weight (72.05 ± 6.96 kg vs. 73.50 ± 6.68 kg, *p* = 0.034), fat mass (11.99 ± 3.13 % vs. 13.98 ± 3.92 %, *p* = 0.030), body mass index (22.04 ± 0.85 kg/m^2^ vs. 22.49 ± 0.92 kg/m^2^, *p* = 0.049), and mental health component summary score (MCS) of the questionnaire SF-36 (53.95 ± 3.47 vs. 58.33 ± 4.50, p = 0.044). Scores on the general health (77.88 ± 14.56 vs. 89.75 ± 13.76, *p* = 0.025) and mental health (81.50 ± 9.30 vs. 90.00 ± 11.71, *p* = 0.018) subscales of the SF-36 also increased significantly.

**Conclusion:**

The COVID-19 lockdown led to an increase in body composition parameters and showed an improvement in the MCS and scores on the general and mental health subscales of the SF-36. Physical performance and sleep quality could be maintained during the home training period. These observations may help trainers for future training planning during longer interruptions in soccer training.

## Introduction

In March 2020, the World Health Organization (2020) declared the global spread of SARS-CoV-2 (COVID-19) as a pandemic. Most governments responded by imposing nationwide lockdowns to reduce the spread of the virus. Public life restrictions also affected amateur and professional soccer, as competitions were postponed or suspended and team training was banned (Sarto et al., 2020). Professional soccer players were forced to train at home, usually following home training plans to maintain physical performance. Match-relevant physical performance in soccer includes endurance and muscle strength (Mohr et al., 2015; Castagna et al., 2020). Therefore, coaches are interested in preserving these components during the absence from team training and incorporating respective exercises into home training. We examined these components before and after the home training period using an isokinetic strength test and a treadmill step test.

The limited sport-specific loads in home training could lead to detraining effects and an increased risk of injury (Mohr et al., 2020). For future training planning, it is consequently important to know the effects of home training on soccer players.

Several studies have investigated the effects of COVID-19 lockdown and home training on the physical performance of professional soccer players, but the findings are partly contradictory. Dauty et al. (2021) reported decreased aerobic capabilities in adolescent soccer players, despite supervised home training, which included two cardio-training sessions and two muscle-strengthening sessions per week. Athletes in other sports, such as handball, also experienced decreased endurance performance, despite a home-based strength and endurance program (Fikenzer et al., 2020). In contrast, Grazioli et al. (2020) found no significant effects on cardiorespiratory fitness in professional soccer players after lockdown, compared with measurements after the traditional off-season. Albuquerque Freire et al. (2020) concluded that aerobic exercise at 65–75% of the maximum heart rate could preserve aerobic capacitance during the lockdown. Another study showed that home training with four or five aerobic sessions and two or three strength training sessions improved aerobic fitness in professional soccer players (Rampinini et al., 2021).

In terms of muscle strength, Moreno-Pérez et al. (2020) found reduced hamstring muscle strength in semi-professional soccer players, although the players had trained at home with specific exercises to maintain muscle strength. Grazioli et al. (2020) found no significant effects in hamstring eccentric strength in comparison to measurements after the traditional off-season.

The lockdown has also affected the mental health of athletes, which can negatively impact their training and performance (Mon-López et al., 2020a). Mental health, vitality, social functioning, and role emotional comprise the mental health component summary score, which represents one dimension of the Short-Form 36 Health Survey (SF-36) that we used.

Studies that conducted online surveys among athletes from different sports in Sweden and South Africa during the lockdown found negative effects on athletes' mental health, such as feelings of depression (Håkansson et al., 2020; Pillay et al., 2020), lack of energy, and lack of motivation to exercise (Pillay et al., 2020). In contrast, a study of professional soccer players showed that mental scores stayed stable during lockdown (Dauty et al., 2021), and another study even showed that soccer players felt psychologically better during this period because they had more time to spend with family and friends (Paravlic et al., 2022).

Sleep is as well important for athletes' physiological and psychological states (Dickinson and Hanrahan, 2009; Erlacher et al., 2011), so we used the Pittsburgh Sleep Quality Index (PSQI) to subjectively assess sleep quality. Reduced sleep quality can have a negative impact on an athlete's performance (Andrade et al., 2016). Some studies have shown, that the sleep quality of athletes deteriorated during the lockdown and the number of hours of sleep increased (Mon-López et al., 2020a,b; Romdhani et al., 2021). Other studies did not find significant changes in athletes' sleep quality during the lockdown (da Silva Santos et al., 2021; Soares et al., 2021).

Keeping daily routines (Pensgaard et al., 2021) and maintaining physical activity may lead to improvements in sleep quality (Irandoust and Taheri, 2017; Sañudo et al., 2020; Trabelsi et al., 2021a) and mental health (Chen et al., 2020; Senişik et al., 2021; Wright et al., 2021) in general and during the lockdown.

To the best of our knowledge, few studies had conducted baseline measurements of players immediately before the start of the lockdown, and few studies provided monitored home training for players during the lockdown. We compared measurements performed 10 days before the lockdown was announced with measurements taken after 13 weeks of monitored home training. Most studies that examined the physical performance and mental health during the lockdown focused on adults and few on youth soccer players, although mental health disorders are generally more prevalent in youth (Kieling et al., 2011; Zhou et al., 2020).

This study aimed to investigate the effects of 13 weeks of monitored home training on physical performance, sleep quality, health-related quality of life, and body composition parameters on professional youth soccer players during the 2020 COVID-19 lockdown in Germany.

## Materials and Methods

### Participants

This study involved eight professional youth soccer players (age range 16–19) belonging to a U19 Bundesliga team from Hesse, Germany. This league is the highest division in professional German soccer. The mean body weight was 72.05 ± 6.96 kg and the height was 1.81 ± 0.07 m. The exclusion criteria were (a) cardiovascular disease, (b) disease of the respiratory tract, (c) an acute infection, and (d) a current injury or persisting sequelae from a prior lesion.

All the players gave their written informed consent to participate in the study. For participants aged under 18 years, parental consent was also obtained. The originally planned study was approved by the Frankfurt am Main University Ethics Committee. The data set of this study yielded the research question presented here.

### Experimental Design

The initial aim of this study was to determine the effects of a new training device on youth soccer players, intending to establish it in professional soccer. The study design was such that half of the subjects (*n* = 10) underwent training using the invention over a 3-week period in addition to conventional soccer training, while the other half of the subjects were to form the control group. To determine differences between the experimental and control group, measurements were to be made before and after the invention. The first measurements for this study took place in early March 2020 in a physiotherapy and training center. The subjects passed through different stations one after the other. First, height was measured, and a bioelectrical impedance analysis was performed. After the subjects warmed up on the bicycle for 5 minutes, the isokinetic strength measurements of the extensors and flexors of the knee were obtained. Finally, the step test on the treadmill including the measurement of lactate on the earlobe followed. After completion of the last measurements, the players completed two questionnaires to assess subjective sleep quality (PSQI), and health-related quality of life (SF-36).

By mid-March, half of the subjects would have performed interval training with the training device in addition to the conventional training. However, due to the Corona pandemic and associated contact restrictions, training on the device as well as conventional soccer training could not be continued, so all players of the U19 team had to train at home with assigned training plans from mid-March to mid-June. As the Corona pandemic measures were relaxed from June onwards, a renewed attempt was made to conduct the study. Again, initial measurements of 20 youth soccer players from the U19 Bundesliga team took place, with eight subjects having already participated in the first measurement in March. Except for a change in the execution of the step test on the treadmill, the procedure was identical to that in March. Data from the eight subjects who participated in the March and June measurements were analyzed for this research question.

### Home Training

The home training period in the present study lasted a total of 13 weeks. The start of home training was marked by the declaration of the COVID-19 outbreak as a pandemic and the accompanying contact restrictions. Since the COVID-19 measures were relaxed from June 2020, the home training period could be ended in mid-June 2020.

To maintain the players' performance during this period, coaches assigned home training schedules to the players. An example of a typical 1-week training program during the lockdown is shown in Table 1. Players were instructed to train 5–6 days per week. Each training session consisted of a warm-up, exercises for strength, agility, or stability, and endurance training as a substitute for soccer training. Before the lockdown was proclaimed, the players trained twice a week at high intensity, including a day of competition, twice a week at medium intensity, and 2 days regeneratively (Table 2). This training structure was maintained during the lockdown period. High-intensity interval training (HIIT) was carried out on days with a high training load, and low-intensity training included cycling and running for 30–45 minutes. The exercises varied weekly, but the load levels remained the same. Technological devices such as tracking apps were used to quantify the soccer players' training load and training values (e.g., heart rate). Video training also assisted in monitoring the execution of the training. The following materials were used for the home training: jump rope, tape, beverage crate, soccer, and a tennis ball.

**Table 1:** An example of a typical home training schedule of professional youth soccer players during the COVID-19 lockdown.

**Monday**	**Tuesday**	**Wednesday**	**Thursday**	**Friday**	**Saturday**
**Warm-up** Each 3×1 min, 1 min rest rope skipping in motion running: 1. Right leg forward in the rhythm of two 2. Left leg forward in the rhythm of two 3. Rhythm of three 4. On the spot with an intermediate jump 5. On the spot without an intermediate jump	**Warm-up** Prevention	**Warm-up** Prevention	**Warm-up** Prevention	**Warm-up** 4×30 sec, 30 sec rest rope skipping	**Warm-up** Prevention
**Exercises** Stabilizing roller 35 sec, 10 sec rest	**Exercises** Mobility program, scroll, stretch	**Exercises** Standing scale each 3×5/side; Front knee bend with water crate 3×8; Lungeing steps dynamic walking each 3×6; Hip thrust 3×6 on tiptoe and with a mini band; Burpees 3×6; Alternate jumps each 3×6	**Exercises** Upper body circle 3×: plank to push-up 12×; Side support pelvis lift and lower 10×; Breast float in probe position 12×; Push-up 1× plus climber 3×12; Rowing at the table max.; Russian twist 20×; Pelvic lifting 20×; Push-up with raised legs	**Exercises** Standing scale each 3×5/side; Front knee bend with water crate 3×8; Lungeing steps dynamic walking each 3×6; Hip thrust 3×6 on tiptoe and with a mini band; Burpees 3×6; Alternate jumps each 3×6	**Exercises** Mobility program, scroll, stretch
**Endurance^a^** 1. Prevention^b^ 2. Uphill runs 8×20 m with a ball on 90%, 30 sec rest 3. Dribbling course: slalom/finten/holding up the ball 2×10 min, 2 min rest	**Endurance** 1. 5 min running ABC 2. 8×15 m sprints 100%, each 10 sec rest, 4 min juggling break with the ball; 8×15 m sprints 100%, each 10 sec rest 3. Run	**Endurance** Cycling 40 min	**Endurance** 1. Preventio n2. Uphill runs 8×20 m with a ball on 90%, 30 sec rest 3. Dribble course: slalom/finten/holding up the ball 2×12 min, 2 min rest	**Endurance** 1. 5 min running ABC 2. 7×1 min HIT^c^ runs 80-90%, each 2:30 min trotting break 3. 10 min run out	**Endurance** Endurance run 45 min

a *The color represents the intensity load (red, high intensity; orange, medium intensity; green, regenerative)*.

b *Supine position, knees, and hips bent 90 degrees, feet planted, hip rolls, 5×/side; supine position, knees stretched in the air, legs spread and tightened, 5×/side; supine position, knees stretched in the air, hip rolls, each 5×/side; lateral position, pelvis pressed forward, upper leg raised and lowered, each 5×/side; prone position, arm in U-position, the knee is brought to elbow alternately, 5×/side; forearm-to-instep, 5×/side; supine position, legs stretched and spread apart, pelvis lifted as high as possible, 3×3 sec; Glute Bridge, knee brought to chest alternately, 3×3/side; supine position, legs crossed, rotated left/right, 3×/side; prone position, arm, and legs raised, leg bent and extended, 5×/side; Sumo-quat, 5×*.

c *High-intensity training*.

**Table 2:** Overview of the training structure before the COVID-19 lockdown.

**Monday^a^**	**Tuesday**	**Wednesday**	**Thursday**	**Friday**	**Saturday**
Free	Speed strength training Soccer training	Free	Speed strength training Soccer training	Free	Competition
Upper body training Core training Soccer training	Jump training	Free	Upper body training Torso training	Jump training	Free

a *The color represents the intensity load (red, high intensity; orange, medium intensity; yellow, medium/low intensity; green, regenerative)*.

### Body Composition Analysis

The following metrics were considered for this study: height, body weight, body mass index (BMI), fat mass, and muscle mass. A stadiometer (seca 274, seca, Hamburg, Germany) was used to measure height, and the other measurements were taken with a bioelectrical impedance analyzer (seca mBCA 515, seca, Hamburg, Germany). All measurements were taken barefoot. During bioelectrical impedance measurement, subjects were asked to assume an upright posture, bend their knees slightly, and not move during the measurement.

The data was stored pseudonymously in the database of the PC software seca analytics 115.

### Isokinetic Strength

After performing a 5-minute warm-up on a cycle ergometer (kardiomed 521, proxomed, Alzenau, Germany), the subjects were seated on an isokinetic dynamometer (Biodex System 3, Biodex Medical Systems, Inc., New York, USA) in an adjustable chair to assess the unilateral strength of the contraction of the flexors and extensors of the knee. A pelvic strap, two diagonal straps from the shoulder to the contralateral side of the pelvis, and a strap on the examined leg at the level of the ankle were used for fixation. The height of the rotational axis of the dynamometer was set with the knee bent to 90 degrees. The subjects were instructed to bend and extend their respective knee, starting with the dominant leg, as quickly as possible and with maximum effort. Five repetitions were performed before the sequence was repeated on the knee of the other side. For this, the apparatus had to be slightly modified. The angular speed parameter of 60°/s was used for the measurements. The results were presented in absolute peak torque.

All measured parameters were recorded by the Biodex system and displayed numerically and graphically. The output data was manually transferred to Microsoft Excel. One participant interrupted the exercise due to pain.

### Endurance Performance

The endurance performance was determined by the individual aerobic and anaerobic threshold, and the maximum lactate level in a step test on the treadmill (T7xi, Matrix Fitness, Frechen, Germany). In March, the treadmill's initial speed started at 8.5 km/h with a stepwise increase of 1.5 km/h every 4 minutes. In between, there was a 30-second break, during which the heart rate and lactate level on the earlobe was measured (Sirius Lactate Scout, h/p/cormos, Nussdorf-Traunstein, Germany). To avoid the risk of injury, the players started in June at a speed of 6 km/h with an increase of 2 km/h every 3 minutes and a break of 10 seconds. The step test was performed to the maximum exhaustion of the subjects. If a player did not complete a stage, the speed was calculated based on the minutes run per stage with percentage calculation. The individual anaerobic threshold was determined using the Dickhuths threshold model (Dickhuth et al., 1991).

### PSQI and SF-36

The PSQI is a questionnaire that captures subjective sleep quality over the past month. The questionnaire consists of 19-self-report questions and five other-report questions that can be answered by the partner or roommate. These five questions are not included in the evaluation and were not identified in this study. The 19 questions can be assigned to seven components (subjective sleep quality, sleep latency, sleep duration, sleep efficiency, sleep disturbance, medication intake, daytime dysfunction). Each component can take a value from 0 to 3 in the evaluation. The total score is the sum of the component scores, ranging from 0 to 21, with higher scores representing worse sleep quality (Buysse et al., 1989). One questionnaire was incompletely filled in.

The SF-36 is an instrument to measure health-related quality of life over the past 4 weeks with 36 items and eight scales: physical functioning, role physical, bodily pain, general health, vitality, social functioning, role emotional, and mental health.

For each item, scores were coded, added up, and then converted into a scale ranging from 0 to 100, with 100 representing the highest possible health status (Jenkinson et al., 1993). After using the German standard mean values, standard deviations, and regression coefficients (1994), and transforming these values, the PCS and the MCS resulted (Morfeld et al., 2011).

Both questionnaires were scored by hand and in Excel, according to the regulations of the manual.

### Statistical Analysis

The data was processed with Excel. Statistical analysis was performed using the software program R (version 4.0.3). The normal distribution of the pre- and post-test differences for each variable was assessed using the Shapiro-Wilk test. Comparison of pre-and post-measurements was performed depending on distribution using a paired *t*-test (body mass, BMI, muscle mass, isokinetic strength measurements, endurance performance, total score of PSQI, PCS, MCS, and the subscales general health, vitality, mental health of the SF-36) or a paired Wilcoxon-Sign-Rank test (height, fat mass, subscales of the PSQI and the subscales physical functioning, role physical, bodily pain, social functioning, role emotional of the SF-36). The significance level was set at 0.05, and all values were reported as mean ± standard deviation (SD). The Effect Size (ES) for the *t*-test was computed using Cohen's D, and the effect size *r* was used for the Wilcoxon-Sign-Rank test. Threshold values for Cohen's D are <0.2, 0.5, and 0.8 for small, medium, and large effects, respectively (Cohen, 1988). For the effect size *r*, threshold values are 0.1 (small effects), 0.3 (moderate effects), and 0.5 (large effects) (Coolican, 2009; Fritz et al., 2012).

## Results

**Figure 1:**
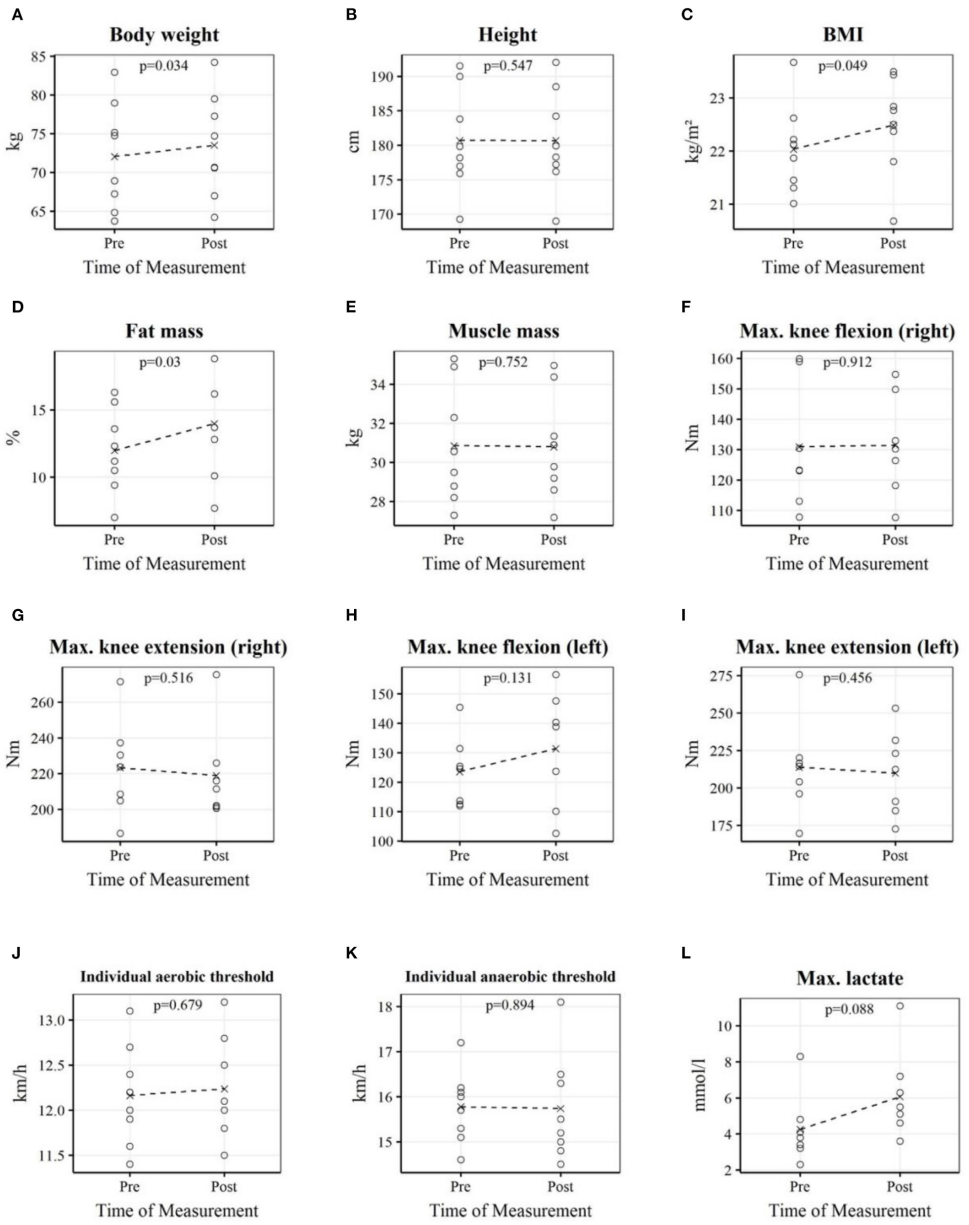
Each plot depicts professional youth soccer players' individual measurements as well as the mean before and after the home training period. Body weight **(A)**; Height **(B)**; BMI **(C)**; Fat mass **(D)**; Muscle mass **(E)**; Max. knee flexion (right) **(F)**; Max. knee extension (right) **(G)**; Max. knee flexion (left) **(H)**; Max. knee extension (left) **(I)**; Individual aerobic threshold **(J)**; Individual anaerobic threshold **(K)**; Max. lactate **(L)**.

In Table 3 and Figure 1, the pre-and post-test measurements of body composition parameters, isokinetic muscle strength, and endurance performance are shown. Body weight, fat mass, and BMI were significantly increased after the home training period due to the COVID-19 lockdown. Muscle mass was not significantly different after the home training period. There were no significant effects on physical performance expressed by isokinetic strength and endurance performance.

**Table 3:** Measurements before and after the home training period.

	**Variables**	***N***	**Pre**	**Post**	***P***	**ES**
Body composition	Body weight (kg)	8	72.05 ± 6.96	73.50 ± 6.68	0.034^*^	−0.93
	Height (m)	8	1.81 ± 0.07	1.81 ± 0.07	0.547	0.25
	BMI (kg/m^2^)	8	22.04 ± 0.85	22.49 ± 0.92	0.049^*^	−0.84
	Fat mass (%)	8	11.99 ± 3.13	13.98 ± 3.92	0.030^*^	0.79
	Muscle mass (kg)	8	30.86 ± 3.03	30.80 ± 2.73	0.752	0.12
Isokinetic strength	KF right (Nm)	7	130.94 ± 20.82	131.44 ± 16.59	0.912	−0.04
	KE right (Nm)	7	223.27 ± 27.27	218.96 ± 26.51	0.516	0.26
	KF left (Nm)	7	123.57 ± 12.22	131.37 ± 19.87	0.131	−0.66
	KE left (Nm)	7	213.79 ± 32.17	209.86 ± 28.55	0.456	0.30
Endurance	I.ae.threshold (km/h)	8	12.16 ± 0.56	12.24 ± 0.56	0.679	−0.15
	I.an.threshold (km/h)	8	15.78 ± 0.80	15.74 ± 1.18	0.894	0.05
	Max.lactate (mmol/l)	8	4.25 ± 1.80	6.06 ± 2.30	0.088	−0.70

*Pre- and post-lockdown measurements are presented as mean ± standard deviation. A paired Wilcoxon-Sign-Rank test was used for the variables height and fat mass, and a paired t-test was used for the remaining variables*.

*N, number of participants; ES, effect size; BMI, body mass index; KE/KF, isokinetic peak torque of knee extensors/flexors; I.ae./I.an. threshold; individual aerobic/anaerobic threshold*.

* *p < 0.05*.

**Figure 2:**
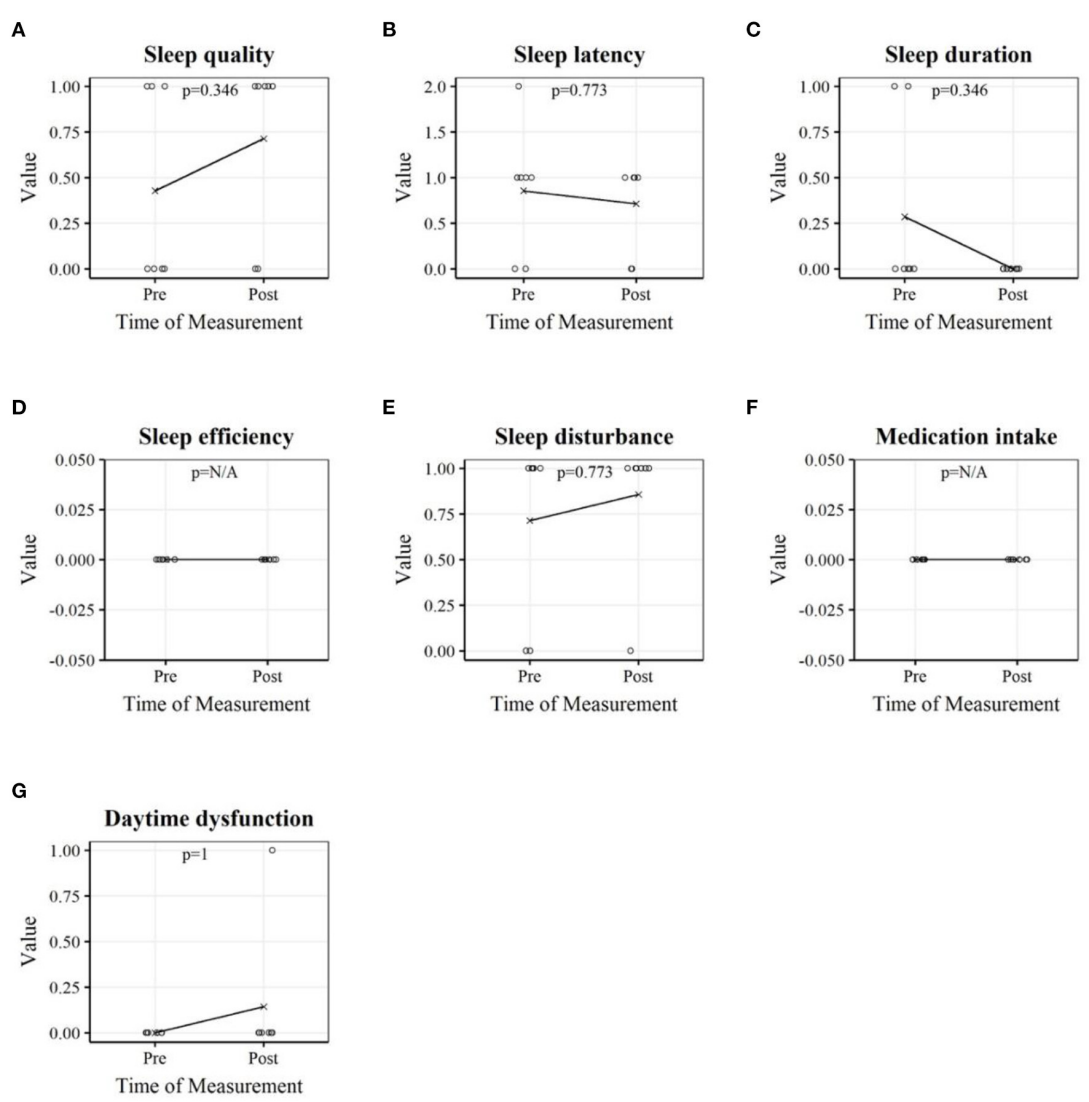
Sleep variables of professional youth soccer players before and after the home training period. Each plot depicts the players' individual measurements as well as the mean. Sleep quality **(A)**; Sleep latency **(B)**; Sleep duration **(C)**; Sleep efficiency **(D)**; Sleep disturbance **(E)**; Medication intake **(F)**; Daytime dysfunction **(G)**.

As can be seen in Table 4 and graphically in Figure 2, neither the total sum score nor the subscales relating to subjective sleep quality changed significantly when comparing the measurements before and after the lockdown.

**Table 4:** Sleep variables before and after the home training period.

**Variables**	***N***	**Pre**	**Post**	***P***	**ES**
Sleep quality	7	0.43 ± 0.54	0.71 ± 0.49	0.346	0.54
Sleep latency	7	0.86 ± 0.69	0.71 ± 0.49	0.773	0.22
Sleep duration	7	0.29 ± 0.49	0.00 ± 0.00	0.346	0.54
Sleep efficiency	7	0.00 ± 0.00	0.00 ± 0.00	NA	NA
Sleep disturbance	7	0.71 ± 0.49	0.86 ± 0.38	0.773	0.22
Medication intake	7	0.00 ± 0.00	0.00 ± 0.00	NA	NA
Daytime dysfunction	7	0.00 ± 0.00	0.14 ± 0.38	1.000	0.38
Total score of PSQI	7	2.29 ± 1.11	2.43 ± 1.13	0.604	−0.21

*Pre-and post lockdown measurements are presented as mean ± standard deviation. A paired t-test was used for the total score of PSQI, and a paired Wilcoxon-Sign-Rank test for the remaining variables*.

*N, number of participants; ES, effect size; PSQI, Pittsburgh Sleep Quality Index*.

**Figure 3:**
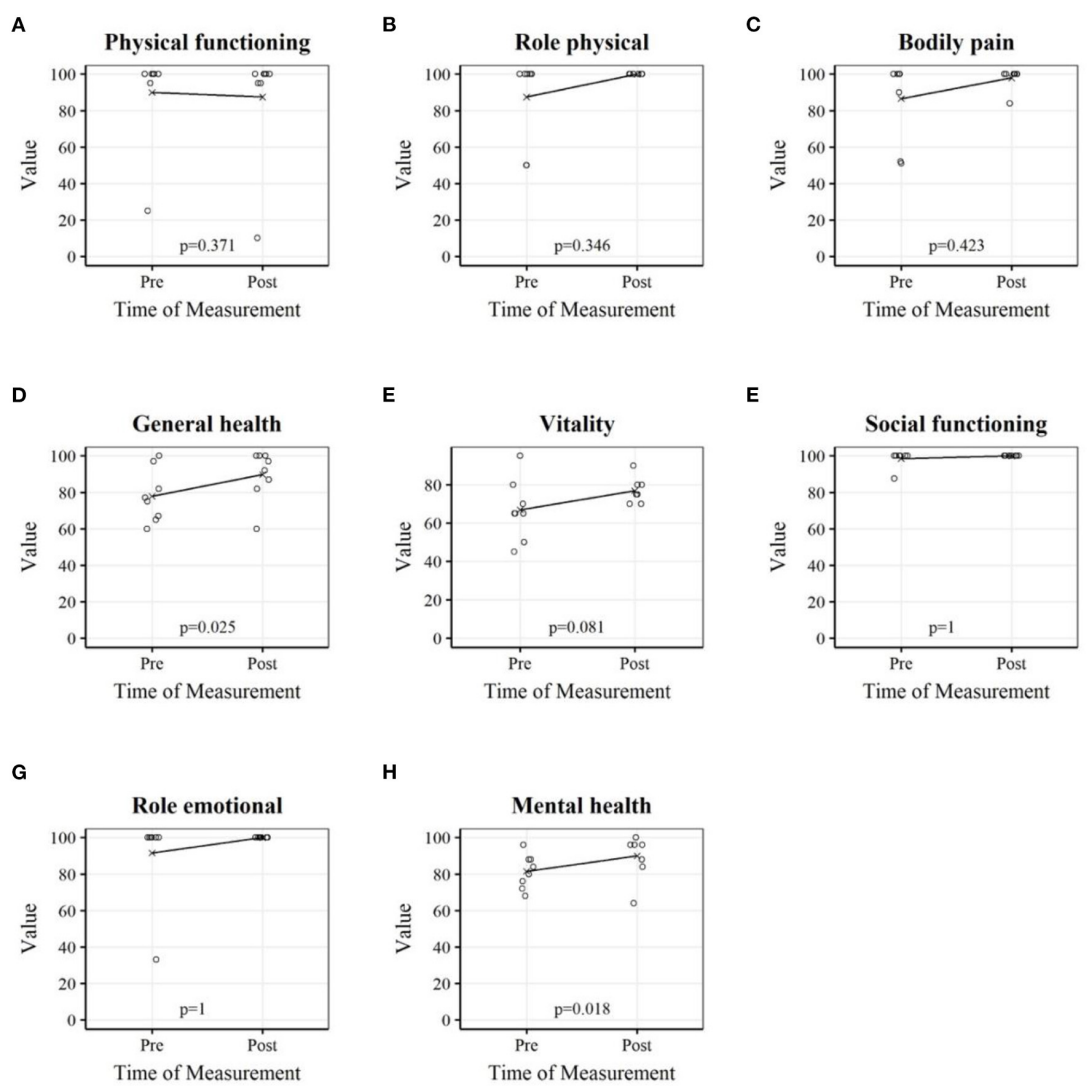
Subscales of health-related quality of life (SF-36) of professional youth soccer players before and after the home training period. Each plot depicts the players' individual measurements as well as the mean. Physical functioning **(A)**; Role physical **(B)**; Bodily pain **(C)**; General health **(D)**; Vitality **(E)**; Social functioning **(F)**; Role emotional **(G)**; Mental health **(H)**.

Table 5 shows the analysis of the subscales and the scales PCS and MCS of the questionnaire SF-36. The MCS and mental and general health subscales showed significant increases after the lockdown (Figure 3).

**Table 5:** Health-related quality of life (SF-36) before and after the home training period.

**Variables**	***N***	**Pre**	**Post**	***P***	**ES**
Physical functioning	8	90.00 ± 26.32	87.50 ± 31.40	0.371	0.50
Role physical	8	87.50 ± 23.15	100.00 ± 0.00	0.346	0.50
Bodily pain	8	86.63 ± 21.95	98.00 ± 5.66	0.423	0.26
General health	8	77.88 ± 14.56	89.75 ± 13.76	0.025^*^	−1.01
Vitality	8	66.88 ± 15.80	76.88 ± 6.51	0.081	−0.72
Social functioning	8	98.44 ± 4.42	100.00 ± 0.00	1.000	0.35
Role emotional	8	91.63 ± 23.69	100.00 ± 0.00	1.000	0.35
Mental health	8	81.50 ± 9.30	90.00 ± 11.71	0.018^*^	−1.09
PCS	8	51.86 ± 8.38	54.13 ± 7.00	0.168	−0.54
MCS	8	53.95 ± 3.47	58.33 ± 4.50	0.044^*^	−0.87

*Pre- and post-lockdown measurements are presented as mean ± standard deviation. A paired t-test was used for the variables general health, vitality, mental health, PCS, and MCS. A paired Wilcoxon-Sign-Rank test was used for the remaining variables*.

*SF-36, Short-Form Health 36; N, number of participants; ES, effect size; PCS, physical health component summary score; MCS, mental health component summary score*.

* *p < 0.05*.

## Discussion

The purpose of this study was to assess the effects of COVID-19 lockdown and associated home training on professional youth soccer players' physical performance, sleep quality, health-related quality of life, and body composition parameters compared to pre-lockdown measurements.

From what we know, a small number of studies have focused on the effects of lockdown on professional youth soccer players.

### Body Composition

Similar to our findings, Grazioli et al. (2020) reported a significant increase in body weight and fat mass in Brazilian professional soccer players after 63 days of home training compared with the regular off-season. The first measurement of the players took place 24 days after a traditional off-season in December 2019, and the second measurement after 63 days of home training in May 2020. During the lockdown, the players performed home-based workouts only using their body weight as resistance. One limitation of that study is that the players were not examined just before the beginning of the home training, whereas in our study a baseline measurement was performed 10 days before the announcement of the lockdown. Another study showed that body fat percentage in professional soccer players was significantly increased after a 7-week lockdown period compared to a 5-week transition period. The training program of the players in this study included muscle strength and power training three times per week and cardiorespiratory training four times per week (Parpa and Michaelides, 2021). Herrera-Valenzuela et al. (2020) also showed increased body weight in combat sports athletes during the lockdown. The athletes answered an online survey about their body weight before and after starting home training. Unlike our study, the combat sports athletes did not have a uniform, supervised home training program.

A Google Forms survey of South African elite and semi-elite athletes conducted in late April 2020 showed that athletes' nutrition deteriorated during the COVID-19 lockdown (Pillay et al., 2020). Poor nutrition may also have had a negative impact on the body composition of our players.

Furthermore, an online questionnaire answered by Spanish professional and non-professional soccer players showed that the lockdown period reduced training intensity and duration (Mon-López et al., 2020a). Another study with elite and sub-elite athletes from different sports has also shown that training frequency and duration decreased significantly during lockdown (Facer-Childs et al., 2021). Reduced physical activity can lead to changes in body composition. By using tracking apps and video training, we tried to counteract this effect.

Some authors compared lockdown with detraining periods of soccer players (Castagna et al., 2020; Paoli and Musumeci, 2020). Detraining is defined as the loss of training-induced adaptions as a result of an insufficient training stimulus (Mujika and Padilla, 2000). In many cases, detraining effects were carried out in the off-season of soccer players, which rarely lasts more than 4–6 weeks (Silva et al., 2016). Generally, the off-season is characterized by two phases: a complete training cessation for a few days and a training phase in which the players have to follow a personally designed training plan. Similar to the off-season, no soccer training took place during the lockdown, and players trained according to training plans. Unlike detraining studies, the lockdown lasted longer and players had no access to professional training equipment. Psychological constrictions associated with lockdown may also have played a role (Mon-López et al., 2020a).

Compared to a detraining study, an increase in fat mass, also measured with a bioelectrical impedance analyzer, was found in professional adult soccer players after an off-season period (6 weeks) with training programs that included 4 weeks of aerobic and strength training sessions (Requena et al., 2017). A significant increase in fat mass was also obtained after an off-season with training programs that included 4 weeks of aerobic and strength conditioning exercises (Sotiropoulos et al., 2009), after 4 weeks of low-intensity aerobic running, three times per week, in a 6-week period (Koundourakis et al., 2014), and after 3 weeks of high-intensity running interval training and strength training in a 5-week detraining program (Suarez-Arrones et al., 2019). Unlike the studies mentioned, Joo (2018), showed no changes in fat mass, assessed by bioelectrical impedance analysis, after a detraining (no physical activity for 2 weeks) and retraining (3 weeks of high-intensity aerobic exercises) period. He surmised that 2 weeks of detraining do not lead to changes in professional soccer players' body composition (Joo, 2018).

All of the above studies were conducted by adults, unlike our study in which the participants were youth athletes where the analysis of the effects can be complicated by simultaneous growth-related increases in the same period (Faigenbaum et al., 2013). However, the height of the players did not change significantly during the lockdown.

### Health-Related Quality of Life and Sleep Quality

At baseline measurement in March 2020, the MCS of the questionnaire SF-36 was higher than the PCS. Both scores increased after the lockdown, with MCS significantly higher in June than in March. Scores on the general and mental subscales also assumed significantly higher scores after the home training period (Figure 3). The mental health subscale refers to general mental health, including, e.g., depression and anxiety, and the general health subscale relates to a personal assessment of health, expectations for the future, and resilience to illness.

Most of the questions in the questionnaire are related to the state of health in the last 4 weeks (Kopjar, 1996).

The MCS includes the subscales vitality, social functioning, role emotional, and mental health.

It is well known that physical activity is associated with mental health (Chekroud et al., 2018).

Concerning the COVID-19 situation, physical activity counteracted the lockdown's negative mental effects (Chen et al., 2020; Senişik et al., 2021; Wright et al., 2021). In general, adolescents showed decreased physical activity and increased screen time during the COVID-19 lockdown (Xiang et al., 2020). Our players differed from their peers in this respect because the training plans allowed them to maintain high levels of physical activity, which may have prevented negative mental health effects during the lockdown. A study by Grimson et al. (2021) showed that physical activity above 250 min per week during lockdown led to a positive impact on mental health in senior professional soccer players.

Furthermore, the training schedules have allowed players to maintain their daily routine, which may also have led to fewer mental health problems (Pensgaard et al., 2021; Dönmez et al., 2022).

Similar to our finding, Dauty et al. (2021) showed that mental scores stayed stable during the lockdown in professional youth soccer players, which could be explained by the fact that the players' training was monitored via the web and a psychological follow-up took place. In our study, the players were also in contact with their teammates and coaches through video training sessions. Another study of professional soccer players reported that players felt better during the lockdown because they were able to spend more time with their family and friends (Paravlic et al., 2022).

Other results showed up in athletes in top leagues of soccer, ice hockey, and handball in Sweden (Håkansson et al., 2020). The COVID-19 lockdown contributed to psychological distress and worries about one's sport and one's future in the sport (Håkansson et al., 2020). Supporting this result, another survey on athletes from South Africa found out that COVID-19 had significant mental effects on athletes, like feelings of depression (Pillay et al., 2020). Only the first mentioned questionnaire included athletes under 18 years of age (Håkansson et al., 2020).

The studies mentioned above consisted of athletes from other countries, where other COVID-19 restrictions may have prevailed.

Other assumptions that could explain our observation are that the double burden of school attendance and high-performance sport was eliminated during the lockdown, allowing the players to focus more on soccer. Furthermore, the pressure to prove oneself for a professional team was removed during this time.

During the lockdown, a significant decrease in mental wellbeing was also predicted by sleep quality (Trabelsi et al., 2021b). We did not observe significant changes in sleep quality as measured by the PSQI in our study. The total score, as well as the scores of the subcomponents of the questionnaire showed no significant changes when answered before and after the home training period. The total score of the PSQI indicated good sleep quality in both cases (Buysse et al., 1989). Figure 2 shows that the scores in the variables sleep quality, sleep disturbance, and daytime dysfunction increased in the measurement after the lockdown compared to the measurement before the lockdown. The values in the variables sleep latency and sleep duration decreased.

A study of 15 youth badminton athletes also found no significant changes in sleep quality and quantity when comparing measurements before the COVID-19 period (July 2019) and during the COVID-19 period (July 2020) (da Silva Santos et al., 2021). Similarly, Soares et al. (2021) could not detect major changes in the sleep quality of elite athletes during the COVID-19 lockdown.

Other studies have shown that the COVID-19 pandemic has led to worsened sleep quality in populations of several countries (Bigalke et al., 2020; Fu et al., 2020; Gualano et al., 2020; Huang and Zhao, 2020; Shillington et al., 2021; Taporoski et al., 2021; Trabelsi et al., 2021a,b). A study using an international Google online survey to forty-one research organizations from different continents found that all PSQI components increased during home confinement compared to before home confinement (Trabelsi et al., 2021a).

It was also found that mainly elderly people were affected by sleep problems (Taporoski et al., 2021). Since we studied young subjects, this might explain our observations.

Furthermore, physical activity not only leads to an improvement in mental health but also in sleep quality (Irandoust and Taheri, 2017; Sañudo et al., 2020; Trabelsi et al., 2021a). As our players exhibited high levels of physical activity during the lockdown, this could also be an explanation for our results.

However, a deterioration in sleep quality was also found in other studies with athletes as subjects. A worsened sleep quality and increased hours of sleep were found in men during the lockdown (Mon-López et al., 2020a). Similar results have been observed in Spanish handball players (Mon-López et al., 2020b) as well as in a study of 3,911 athletes from 49 countries (Romdhani et al., 2021). The authors (Mon-López et al., 2020b) concluded that psychological factors during the lockdown significantly influenced sleep behavior. Our participants showed an improvement in the MCS during the lockdown compared to the baseline measurement. This observation could explain why the players' sleep behavior did not deteriorate, in contrast to these studies.

It must also be taken into account that the players in our study answered the PSQI in June, assessing their sleep quality in May/June. Other studies have assessed sleep quality in the initial stage of the COVID-19 pandemic and thus may have reached different conclusions.

### Physical Performance

Soccer experts expected negative effects on professional soccer players' physical capabilities after the lockdown (Mohr et al., 2020).

We observed no significant changes in the endurance performance of the soccer players. Similar to our observations, Grazioli et al. (2020) also reported no significant changes in cardiorespiratory fitness after the COVID-19 home training in adult professional soccer players. After the off-season period and after the COVID-19 lockdown, the players were tested using an adaption of the Yo-Yo test. They were instructed to perform exercises at home using only their body mass as resistance. Albuquerque Freire et al. (2020) concluded that aerobic training at 65–75% of maximum heart rate maintained aerobic performance during the lockdown. Rampinini et al. (2021) even showed that home training with increased aerobic training volume improved aerobic fitness in Italian professional soccer players.

In contrast, Dauty et al. (2021) showed decreased aerobic capacity using a pre-and post-lockdown Yo-Yo test in 14-year-old elite soccer players. The players performed two cardio-training sessions and two upper and lower limb muscle strengthening sessions per week. Salazar et al. (2020) reported significantly worse results in physical performance after the lockdown in young basketball players, and elite handball players also showed a reduction in endurance capacity (Fikenzer et al., 2020).

At 6 days a week, our players trained more often than the athletes in the studies mentioned above, explaining these discrepancies. In addition, our home training was monitored, in contrast to that of the handball and basketball players.

No changes were observed in isokinetic strength exercises of knee flexion and knee extension. Grazioli et al. (2020) also reported no significant changes in hamstring eccentric strength in male professional soccer players after 63 days of home training compared to tests after the traditional off-season period. Another study found that in professional soccer players, in whom home training was controlled by video training, no changes in isokinetic peak torque were noted in contrast to the group of soccer players who were not controlled (Scoz et al., 2022). In contrast, another study showed reduced eccentric hamstring strength in soccer players in a 7-week home training period despite a training program that included strength-based activities (Moreno-Pérez et al., 2020).

A detraining study came to similar conclusions to ours. They measured the isokinetic strength of the lower limbs after a 4-week detraining period and then followed by training for 4 weeks with exercises for aerobic capacity and maximal strength. Neither the training cessation nor the training program affected the isokinetic strength performance of elite youth soccer players (Vassilis et al., 2019). In youths, neural adaptations play a major role in training-induced strength gains (Behm et al., 2008; Faigenbaum et al., 2009, 2013). When youths are active, participating in activities that are not specifically resistance exercises, there may be efficient stimuli to maintain the training effects (Chaouachi et al., 2019).

### Limitations and Conclusion

This work has some limitations that should be acknowledged. First, we had a small sample size. Second, the nutrition of the players during the home training period was not controlled. These two limitations were because the COVID-19 pandemic was an unpredictable development of indeterminate duration. A pre-planned study was not possible. Fortunately, the first measurement was performed immediately before the announcement of the lockdown. All available data is essential because this situation was unique and cannot be replicated to the same extent in the future. Third, we examined professional soccer players in our study, and this sample of participants did not allow us to form a control group.

In the case of another lockdown with prior planning, one could control the subjects' nutrition and add growth hormones as a measurement constant to describe the influence of home training on it.

In conclusion, the monitored home training schedule was effective in maintaining muscle strength, endurance performance, and sleep quality during the COVID-19 lockdown. The player's body weight, BMI, and fat mass increased significantly, just as the MCS and the general and mental health subscales of the questionnaire SF-36.

These observations are important for training planning when players return to their regular training after 13 weeks of home training. It may help trainers create individual training programs for professional youth soccer players in longer breaks of interruption of soccer training or in case of another lockdown.

## Data Availability Statement

The raw data supporting the conclusions of this article will be made available by the authors, without undue reservation.

## Ethics Statement

The studies involving human participants were reviewed and approved by Ethikkommission Fachbereich 5 Psychologie und Sportwissenschaften, Goethe-Universität Frankfurt am Main. Written informed consent to participate in this study was provided by the participants' legal guardian/next of kin.

## Author Contributions

JK: investigation, data collection, analysis and interpretation of the findings, performed the statistical analysis, and writing the original draft. JS: investigation, data collection, analysis and interpretation of the findings, review, and editing. FP: designed the experiments and review. WB: designed the experiments, review, and editing. All authors contributed to the article and approved the submitted version.

## Conflict of Interest

FP was employed by Eintracht Frankfurt Fußball AG. The remaining authors declare that the research was conducted in the absence of any commercial or financial relationships that could be construed as a potential conflict of interest.

## Publisher's Note

All claims expressed in this article are solely those of the authors and do not necessarily represent those of their affiliated organizations, or those of the publisher, the editors and the reviewers. Any product that may be evaluated in this article, or claim that may be made by its manufacturer, is not guaranteed or endorsed by the publisher.
